# DS-ARP: A New Detection Scheme for ARP Spoofing Attacks Based on Routing Trace for Ubiquitous Environments

**DOI:** 10.1155/2014/264654

**Published:** 2014-08-27

**Authors:** Min Su Song, Jae Dong Lee, Young-Sik Jeong, Hwa-Young Jeong, Jong Hyuk Park

**Affiliations:** ^1^Department of Computer Science and Engineering, SeoulTech, Seoul 139-743, Republic of Korea; ^2^Department of Multimedia Engineering, Dongguk University, Seoul 100-715, Republic of Korea; ^3^Humanitas College, Kyung Hee University, Seoul 130-701, Republic of Korea

## Abstract

Despite the convenience, ubiquitous computing suffers from many threats and security risks. Security considerations in the ubiquitous network are required to create enriched and more secure ubiquitous environments. The address resolution protocol (ARP) is a protocol used to identify the IP address and the physical address of the associated network card. ARP is designed to work without problems in general environments. However, since it does not include security measures against malicious attacks, in its design, an attacker can impersonate another host using ARP spoofing or access important information. In this paper, we propose a new detection scheme for ARP spoofing attacks using a routing trace, which can be used to protect the internal network. Tracing routing can find the change of network movement path. The proposed scheme provides high constancy and compatibility because it does not alter the ARP protocol. In addition, it is simple and stable, as it does not use a complex algorithm or impose extra load on the computer system.

## 1. Introduction

Ubiquitous computing has been perceived as the new paradigm for a comfortable life in recent times. In particular, the use of smart applications has greatly increased. However, a growing number of security concerns in this environment have emerged at the same time. Ubiquitous environments always need connected networks. Therefore, one of the important considerations in ubiquitous environments is network security. A secure network can maintain rich ubiquitous environments. Therefore, security considerations in the ubiquitous networks are required.

An ARP spoofing attack is an attack in which the media access control (MAC) address of a computer is masqueraded as that of another. Although various studies have addressed ARP spoofing attack detection, and presented countermeasure plans, there are numerous fundamental difficulties in finding an optimized solution. Countermeasure schemes, such as the cache table static management, S-ARP, T-ARP, and ARP Table server synchronization method, have been presented. Nevertheless, because of various reasons, such as compatibility with the existing network configurations, protocols, and administrative overhead, applying these schemes poses difficulties. In this paper, we present a new detection scheme for ARP spoofing attacks using local network information and a routing trace, which can protect the internal network from ARP spoofing. The detection is composed of ARP Cache Table periodic surveillance and Routing trace. Additional network configuration and protocol change are not required in the proposed scheme, since it does not increase the system and network overhead.

The remainder of this paper is organized as follows.  Section 2 discusses related studies including problems in ARP spoofing, security requirements, and existing research.  Section 3 introduces a new detection scheme for ARP spoofing using a routing trace. Finally, Section 4 presents concluding remarks and briefly discusses the scope for further research.

## 2. Related Work

**Figure 1:**
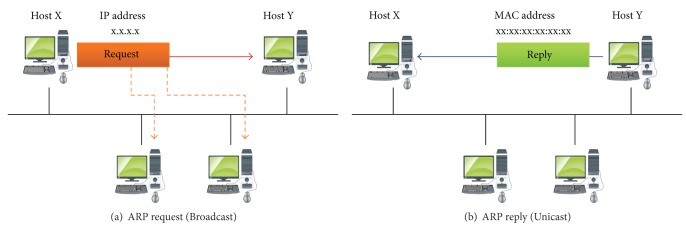
ARP request/reply protocol.

ARP is used to map the MAC physical address and the IP network address, as presented in Figure 1. When a host on a local network wants to know the MAC address of the corresponding IP address, it broadcasts an ARP request message to all the hosts connected to the network. All hosts receive the ARP request, and the host with the information on the requested IP address and MAC address will reply with the *〈*IP, MAC*〉* pair information via unicast [1].

This *〈*IP, MAC*〉* pair information is efficiently managed through the ARP cache table. Typically, ARP cache entries expire after 20-minute intervals, and in some operating systems, the expiration timer is reset. As shown in Figure 1, Host Y sends an ARP request message to all the hosts connected to the local network to get the MAC address of Host X. Host X sends the ARP reply message, its *〈*IP, MAC*〉* pair information, to Host Y via unicast [2, 3].

### 2.1. Problems in ARP and ARP Spoofing

As ARP updates the host's ARP cache table in the absence of reliable mutual agreement procedures while transmitting the request/reply messages, it has a few fundamental security problems. ARP spoofing attacks are described as follows [4].

- Block host: an attacker, using the ARP spoofing technique, can change the ARP cache table. The packets sent by the host, in which the ARP cache table is changed, do not reach the real destination address but reach the attacker. Thus, the host network can be blocked by the attacker.
- Host impersonation: an attacker can impersonate a host, and, by doing so, can discard the host's packet and cancel the host's request.
- Man-in-the-middle (MITM) attack: an attacker can change the ARP cache table of two hosts and monitor the communication between them.

### 2.2. Security Requirements for an Ideal Solution

We can consider the following security requirements as the response scheme for ARP spoofing attacks [3].

1. Management costs of hosts should be controlled.
2. The cryptographic processing, which can lower the performance of ARP, should be minimized.
3. Prevention and block should be detected with timely warnings, which will alert the administrator about the attack situation.
4. The solution has to be universal and easily applicable.
5. Hardware costs should be minimized.
6. The solution has to be compatible with ARP.
7. It should not slow down the ARP request/reply communications.
8. If possible, it should consider all the ARP attacks.
9. The network traffic should be contained.

### 2.3. Existing Research

Gouda and Huang [5] proposed a structure that resolves the security problems of MAC address and IP address within the Ethernet. The proposed scheme consists of a security server whose two protocols, Invite-accept and Request-reply, are connected to all hosts. Each host checks, through Invite-accept and Request-reply, the IP address and MAC address from the security server, thereby enhancing security [5]. Secure ARP (S-ARP) uses the public and private key pair, authorized through an asymmetric encryption algorithm. All hosts create public and private key pairs during the initial contact of the network and send them with signed certificates to the authoritative key distributor (AKD). Thus, an ARP spoofing attack can be prevented by identifying whether the transmitted request is from a valid user [6]. The port security scheme proposes a form of security using a physical port at the MAC address formed at the switch. Port security can be an effective defense against an attacker who replicates a MAC address. This scheme can definitely protect hosts from MAC-hijacking-type attacks [2, 3]. Dynamic ARP inspection (DAI) is a scheme that can only be used with certain switch types. DAI solves security problems by preventing invalid or malicious ARP packets that are delivered from the network. It identifies whether the ARP packet is valid by comparing the packet at the switch, before it is delivered. If a security problem is detected, the packet is deleted [2, 3]. The ticket-based address resolution protocol (T-ARP) defends ARP spoofing by distributing the centrally secured IP address and MAC address mapping proof. This proof, called ticket, is delivered to the client when it approaches the network. Unlike other public key methods, this protocol can reduce costs [7]. Xing et al. proposed a scheme to capture and filter ARP packets using the WinPcap library. This method involves collecting ARP packets through a WinPcap filtering setting. If the collected ARP packet has a valid value, the ARP cache table is updated [8]. Ramachandran and Nandi proposed a scheme for detecting ARP spoofing through the mismatch of the ARP request/response packet and the *〈*IP, MAC*〉* pair of TCP SYN. When the information in an ARP request/response packet is different from that of the *〈*IP, MAC*〉*, and the TCP connection is made, ARP spoofing is assumed. Following this, all attack packets are dropped and reported to the server [9]. P-ARP proposed by Limmaneewichid and Lilakiatsakun [10] modifies only the confirmed ARP packet, an ARP protocol 28-byte ARP message with an 18-byte authentication data ARP trailer attached [10]. ASA is set to static all the Link Type of ARP Cache Table. ASA is not use the ARP Filter Driver of Kernel layer and directly update using encrypted ARP information in ASA agent [11].

## 3. DS-ARP Scheme

In this section, the proposed detection scheme for ARP spoofing attack using a routing trace, namely detection scheme for ARP spoofing (DS-ARP), is discussed in detail.

### 3.1. Architecture

**Figure 2:**
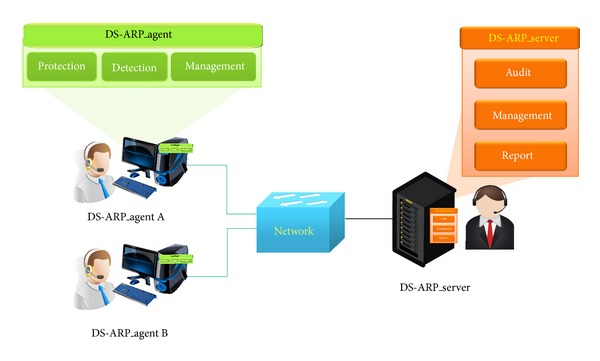
Overall architecture of the DS-ARP scheme.

The architecture of the proposed scheme can be divided into the agent and server side. Detection and protection are the key technologies involved, as shown in Figure 2.

Detection periodically keeps the updated state of the ARP cache table under surveillance. When the ARP cache table is updated, the DS-ARP performs a routing trace to identify the corresponding *〈*IP, MAC*〉* pair information. If an ARP spoofing attack is suspected, it reports to the server and initiates the protection process. It also converts the corresponding *〈*IP, MAC*〉* pair ARP type to static mode.  Table 1 shows the description of acronym.

### 3.2. Operation Process of DS-ARP

The detection module periodically keeps the ARP cache table under surveillance and checks changed items. Once a change in the ARP cache table is identified, the DS-ARP determines whether an ARP spoofing attack has taken place through a routing trace.

The protection module converts the *〈*IP, MAC*〉* pair information, which is changed, by the ARP spoofing attack in the ARP cache table list, to the previous state. It prevents ARP spoofing attacks by changing the link type from a dynamic state to a static state.

**Figure 3:**
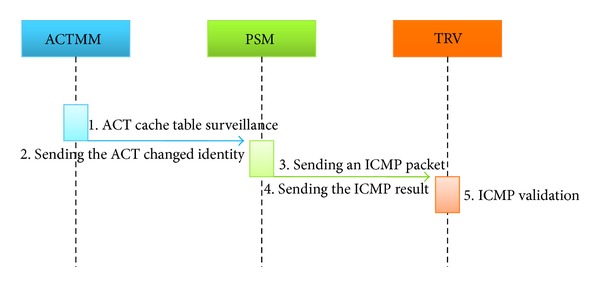
Protocol for the detection stages.

*Step 1 (Detection).* See Figure 3.

① * ACTMM* (ARP cache table surveillance). ACTM periodically monitors the ARP cache table and the information in the host network.
② * ACTMM* →* PSM* (sending the ACT changed identity). The changed information in the ARP cache table is transmitted to PSM using ACTMM.
③ * PSM* (sending an ICMP packet). PSM sends the ICMP packet in which the TTL value is increased to TRV.
④ * PSM* →* TRV* (sending the ICMP result). PSM sends the resultant values of ICMP response to TRV.
⑤ * TRV* (ICMP validation). TRV checks the moving path of the ICMP and identifies ARP spoofing attacks.

**Figure 4:**
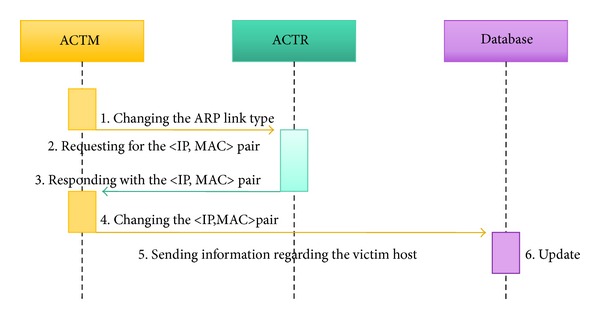
Protocol for the protection stages.

*Step 2 (Protection).* See Figure 4.

① * ACTM* (changing the ARP link type). ACTMN protects the host by changing the ARP link type of the corresponding *〈*IP, MAC*〉* pair from a dynamic mode to a static mode.
② * ACTM* →* ACTR* (requesting for the *〈*IP, MAC*〉* pair). ACTM requests the ACTR for the normal *〈*IP, MAC*〉* pair, that is, before it was changed.
③ * ACTR* →* ACTM* (responding with the *〈*IP, MAC*〉* pair). ACTR responds to ACTM with the normal *〈*IP, MAC*〉* pair before the pair was changed.
④ * ACTM* (changing the *〈*IP, MAC*〉* pair). ACTM applies the normal *〈*IP, MAC*〉* pair to the ARP cache table.
⑤ * ACTM* →* Database* (sending information regarding the victim host). ACTM sends information regarding the victim host to the database.
⑥ * Database* (update). Database updates the information about the victim host to the ARP spoofing attacked state.

### 3.3. Analyses of the Proposed DS-ARP

In this section, we analyze the existing schemes and the proposed DS-ARP based on the security requirements described in Section 2.2. A comparison of current schemes versus the proposed scheme is shown in Table 2.

The scheme of using the WinPcap library by Xing et al. has the disadvantage of continuously monitoring the ARP packets and repeatedly comparing them with local information. The scheme proposed by Ramachandran and Nandi causes excessive traffic in the network. Although S-ARP and T-ARP are the most infallible schemes for preventing ARP spoofing, using a pair of authorized keys, their disadvantage is the requirement of an ARP protocol change. Port security cannot be a perfect solution, as it is vulnerable to MITM attacks.

The DAI has a drawback in that the network configuration and switches need to be changed. Limmaneewichid and Lilakiatsakun proposed an effective scheme that ensures the integrity of the ARP packet. However this scheme slows down the network to an unacceptable level. Abad and Bonilla defined the requirements to be fulfilled by the ARP spoofing solution schemes. Based on these requirements, the existing schemes and the scheme proposed in this study are compared in Table 2. The existing schemes can increase the system and network load. In addition, they either are hardware-dependent or perform limited ARP spoofing detection. Furthermore, the current schemes influence the speed of ARP or result in a direct overhead on the network, and they are not easy to use because of their universal disadvantages. ASA is not compatible with ARP.

The proposed DS-ARP can overcome the problems of the existing schemes and it offers a simple and high-performance solution. The detection and protection scenario for the proposed scheme is discussed in the following two steps.

**Figure 5:**
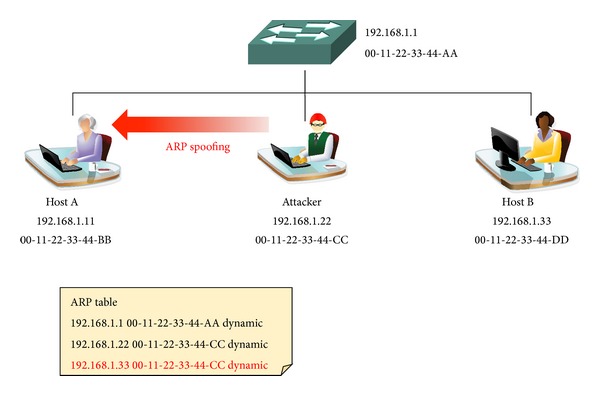
ARP spoofing detection.

*Step 1 (Detection).* When the network is attacked through ARP spoofing, the moving path of the network bypasses the attacker's host and passes the gateway. As shown in Figure 5, if there is a change in the ARP cache table and the gateway of Host A's network information does not move to the primary path through network routing tracing, it can be deemed that an ARP spoofing attack has taken place in the network.

The routing tracing of the network uses the time-exceeded ICMP message of the ICMP protocol. The TTL value of ICMP decreases with each pass through the router. In other words, if the packet is sent after setting the TTL value to one, the first router on the path will cause the time-exceeded message to return. The network path can be traced by increasing the TTL value.

**Figure 6:**
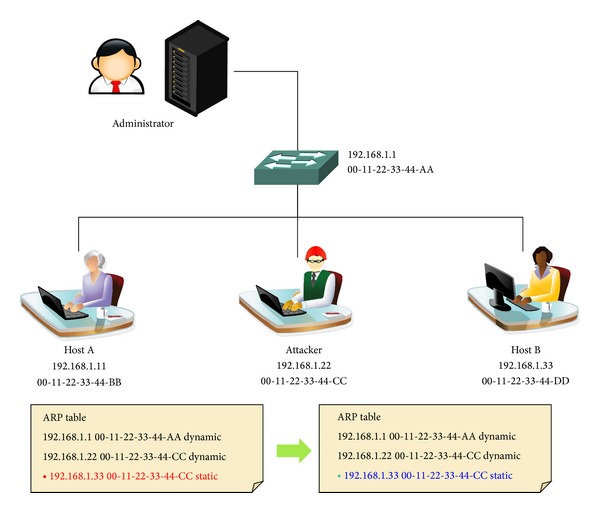
ARP spoofing protection.

*Step 2 (Protection).* Once it is deemed that an ARP spoofing attack has taken place, the changed *〈*IP, MAC*〉* pair of the ARP cache table is restored to that stored in the memory and is converted to static. An ARP spoofing warning is sent to the server.  Figure 6 explains a case of the ARP spoofing detection.

## 4. Conclusion

We discussed a new detection scheme for ARP spoofing attacks based on routing trace for ubiquitous environments in this paper. This scheme detects ARP attacks through real-time monitoring of the ARP cache table and a routing trace and protects the hosts from attackers through ARP Link Type Control which changes from dynamic to static. In addition, it can solve problems such as host impersonation, man-in-the-middle attack, and block of host. And also, the proposed scheme does not require an ARP protocol change or a complex encryption algorithm; moreover, it does not cause high system load.

Despite the various solutions presented in this paper, new attack techniques can still cause new security problems, since the ARP protocol has a few basic weaknesses. Therefore, further studies on resolving the fundamental security vulnerabilities of the ARP protocol are required.

## Figures and Tables

**Table 1:** Description of Acronym.

Acronym	Description
ACTMM	ARP cache table monitor manger
PSM	Packet send manager
TRV	Trace routing validation
ACTM	ARP cache table manager
ACTR	ARP cache table repository
Database	Database

**Table 2:** Comparisons of current schemes versus the proposed scheme.

Classification	T-ARP [7]	Xing [10]	P-ARP [12]	ASA [7]	DS-ARP
Host cost minimization	△	△	△	○	○
Cryptographic technique minimization	△	⊚	△	○	⊚
Warning/detection effectiveness	△	⊚	△	△	⊚
Universality, easy applicability	△	○	○	○	⊚
Hardware costs minimization	○	⊚	⊚	⊚	⊚
ARP compatibility	⊚	⊚	⊚	△	⊚
ARP speed	○	○	⊚	△	⊚
Network loading	⊚	⊚	⊚	⊚	⊚
Security	⊚	○	⊚	⊚	⊚

⊚: Strong, ○: Medium, △: Weak.
